# Physicochemical Characterization and Safety Assessment of Cosmetic Gels and Emulsions Containing Sand‐Extraction Clays

**DOI:** 10.1111/jocd.70517

**Published:** 2025-10-23

**Authors:** Juliana da Silva Favero, Venina dos Santos, Valéria Weiss‐Angeli, Charlene Silvestrin Celi Garcia, Greta Camilla Magnano, João Antonio Pêgas Henriques, Mariana Roesch‐Ely, Diogo dos Santos Miron, Carlos Pérez Bergmann

**Affiliations:** ^1^ University of Caxias do Sul (UCS) Caxias do Sul Brazil; ^2^ Department of Chemical and Pharmaceutical Sciences University of Trieste Trieste Italy; ^3^ Federal University of Rio Grande do Sul (UFRGS) Porto Alegre Brazil

**Keywords:** clays, cytotoxicity, dermal irritability, green formulations, rheological behavior

## Abstract

**Background:**

Clays are widely used in cosmetic formulations for their rheological properties, adsorption capacity, and potential skin benefits. Sustainable sourcing of clays from industrial by‐products, such as sand extraction residues, is gaining interest in the cosmetics industry.

**Aims:**

This study aimed to assess the feasibility of incorporating four clays (I, II, III, IV) derived from sand extraction residues into cosmetic gels and emulsions, evaluating their physicochemical properties, biocompatibility, and dermal safety.

**Patients/Methods:**

The clays were incorporated into two types of hydrophilic vehicles (a nonionic emulsion and a gel). Formulations were characterized for particle size, viscosity, and storage stability over 90 days. Cytotoxicity was evaluated in vitro using HaCaT keratinocytes exposed to clay concentrations up to 1000 μg/mL. Dermal irritation potential was assessed in vivo on 53 healthy volunteers via patch testing.

**Results:**

Clay incorporation affected particle size and viscosity, with gels showing superior physical stability. All clays preserved HaCaT cell viability above 75% at the highest concentration, and in vivo assessments revealed no signs of skin irritation or sensitization. Clay III showed the largest surface area, improving skin adhesion and spreadability, while consistency between in vitro and in vivo data confirms the biocompatibility and cutaneous safety of the formulations.

**Conclusions:**

Clays from sand extraction residues are safe, stable, and biocompatible ingredients for cosmetic formulations. Their incorporation into gels and emulsions provides favorable rheological and dermatological properties, supporting their potential as sustainable raw materials for cosmetic and dermatological applications.

## 1 Introduction

Clays are naturally occurring materials composed of fine‐grained minerals, typically with particle sizes smaller than 2 μm, and they exhibit plasticity when mixed with an appropriate amount of water [1, 2]. Historically used in ceramics, clays have increasingly attracted attention for their versatility in industrial applications, including agriculture, construction, lubricants, fertilizers, and environmental remediation [3, 4]. Their colloidal dimensions, large specific surface area, and ion‐exchange capacity endow clays with rheological, adsorption, and stabilizing properties, making them suitable for use in both pharmaceutical and cosmetic products [1, 5, 6]. In cosmetics, clays are widely applied for their ability to promote skin cell renewal, absorb impurities, remove excess sebum, and reduce visible signs of aging [7, 8, 9, 10, 11]. They are also valued for their cleansing, moisturizing, astringent, and soothing effects, supporting their incorporation into facial masks, emulsions, and cleansers [12, 13]. Specific cosmetic applications include the treatment of acne, seborrhea, and oily skin due to their lipid‐absorbing capacity [14, 15]. Moreover, clays have been increasingly recognized in dermatology for their beneficial effects in managing inflammatory skin conditions such as acne vulgaris, seborrheic dermatitis, and atopic eczema, owing to their adsorptive and anti‐inflammatory properties [16, 17, 18]. Positive effects have also been demonstrated in lesioned skin [19, 20, 21]. These properties make clays suitable both as active ingredients and as excipients in therapeutic topical formulations, enhancing skin barrier function while minimizing the risk of irritation [15, 22, 23]. In pharmaceutical applications, clays serve both as active ingredients or excipients, acting as binders, desiccants, emulsifiers, thickeners, or carriers in oral and topical formulations [1, 5, 6, 24, 25]. The chemical and mineral composition of natural clays varies depending on their geological source and may include quartz, feldspars, carbonates, sulfates, and oxides of iron, aluminum, or titanium [1]. However, certain components such as crystalline silica require strict control due to their potential toxicological effects. Notably, silica levels should remain below 0.1% to minimize carcinogenic risk [1]. Additionally, the high surface area and ion‐exchange properties of clays may result in the adsorption of environmental contaminants such as heavy metals and radionuclides, which may pose safety concerns when used topically [1]. Consequently, pharmacopeias require extensive quality and safety testing, including mineral identification, water content, pH, microbial limits, acid‐soluble substances, and the quantification of impurities such as trace metals and radionuclides when clays are used in pharmaceutical or dermocosmetic formulations [26]. Several studies have explored the potential medical and cosmetic use of natural clays. For instance, Silva et al. [26] investigated the chemical profile of different clays and found higher radionuclide levels in green clay compared to white clay, although their overall mineral composition was comparable. These findings highlight the importance of toxicological screening, particularly for ingredients intended for dermal application. Topical products must be evaluated for skin compatibility, as their ingredients may cause adverse reactions ranging from mild irritation to allergic or systemic responses. Symptoms can include eczematous contact dermatitis, urticaria, acne, and blemishes [27] and may arise immediately or after prolonged use [28, 29, 30, 31]. Common irritants include α‐hydroxy‐acids, propylene glycol, alcohol, fragrances [32, 33, 34] and certain emulsifiers such as PEGs, stearic acid derivatives, and ceteareth compounds [35, 36]. Reducing the number and concentration of such components is a key strategy to improve skin tolerance [37]. Standardized tests such as the patch test are widely used to evaluate the safety of cosmetic products [28, 37]. However, it is important to study the toxicity of the product and/or raw materials in order to assess the potential risk that the ingredients of the formulations may cause to humans [38]. In recent years, the cosmetic and pharmaceutical industries have increasingly embraced the principles of sustainability and circular economy, aiming to minimize environmental impact while maximizing resource efficiency [39, 40]. Within this framework, the reuse of mineral residues such as clays derived from sand extraction processes represents a promising strategy for developing eco‐friendly formulations [41, 42, 43]. Most clays used in cosmetics are primary clays obtained from soil decomposition and typically commercialized in powder form. Their production involves mining operations carried out in preselected areas; once the quarry is cleared, the clay is extracted and transported to a milling plant [44]. However, this extraction process can cause significant environmental impacts, including changes in topography, erosion, deforestation, alterations in water pH, air pollution, and the loss or disruption of terrestrial and aquatic ecosystems, as well as landscape degradation [45]. In response to these issues, recent research has investigated alternative approaches to reduce the environmental burden of clay extraction by treating and purifying mineral residues, with the aim of applying them in cosmetic and pharmaceutical formulations [46]. These by‐products, often discarded as waste, may possess physicochemical characteristics comparable to those of commercial‐grade clays, provided that appropriate purification and safety assessments are conducted [47]. Their valorization not only reduces the ecological footprint associated with raw material sourcing but also contributes to waste management and cost‐effectiveness. The primary aim of this study was to characterize cosmetic gel and emulsion formulations containing four different types of clays (I, II, III, and IV) derived from sand extraction residues, focusing on their physicochemical properties and storage stability. The secondary aim was to evaluate the cutaneous safety of these clay‐based formulations through in vitro cytotoxicity testing on HaCat keratinocytes and in vivo dermal irritation assessments in order to confirm their biocompatibility and suitability for topical and cosmetic use.

## 2 Materials and Methods

### 2.1 Materials

All chemicals were of analytical grade. Four different samples of clay designated as clay I, clay II, clay III, and clay IV, from the sand extraction for the construction of mining companies in the interior of the state of São Paulo, Brazil, were tested in the cosmetic formulations. The samples were decontaminated and characterized according to the described procedure reported in Favero et al. [46]. Before use in the formulations, all clays were decontaminated at the Laboratory of Cosmetics and Drugs Quality Control Laboratory of the University of Caxias do Sul (Caxias do Sul–Rio Grande do Sul, Brazil). The methodology was adapted from the United States Pharmacopeia (2006). This procedure minimized contaminants while preserving the physicochemical properties of the clays. They were selected based on their different physicochemical properties, as previously characterized. To provide a clear overview of their distinguishing features, a summary of their characterization is presented in Table 1.

**TABLE 1:** Physical and chemical characterization of clays I, II, III, IV [46].

Clay	Chemical composition^a^ oxide (wt%)	XRD	Particle size diameter (μm)	Surface area (m^2^/g)
I	Fe 7.5; Si 48.1; Al 28; Mn 0.3; S 0.1; K 0.3; Ca 0.1; Ti 1.2; Mg 0.1	Kaolinite [Al_2_Si_2_O_5_(OH)_4_]	3.6	28.74
II	Fe 4.6; Si 53.4; Al 27.5; Mn 0.1; S 0.1; K 3.1; Ca 0.1; Ti 0.4; Mg 0.1	Kaolinite [Al_2_Si_2_O_5_(OH)_4_]; Ilite (K, H_3_O)(Al,Mg,Fe)_2_(Si, Al)_4_O_10_[(OH)_2_,H_2_O]; Smectite (½ Ca, Na)_0.7_(Al,Mg,Fe)_4_[(Si,Al)_8_O_20_](OH)_4_ · nH_2_O	24.1	22.80
III	Fe 5.1; Si 55.8; Al 26; Mn 0.1; K 3.1; Ca 0.1; Ti 0.6; Mg 0.2	Kaolinite [Al_2_Si_2_O_5_(OH)_4_]; Ilite (K,H_3_O)(Al,Mg,Fe)_2_(Si,Al)_4_O_10_[(OH)_2_,H_2_O]	6.9	38.57
IV	Fe 2.2; Si 54.6; Al 26; K 3.5; Ca 0.6; Ti 0.8; Mg 0.1	Kaolinite [Al_2_Si_2_O_5_(OH)_4_]; Ilite (K,H_3_O)(Al,Mg,Fe)_2_(Si,Al)_4_O_10_[(OH)_2_,H_2_O]	9.7	30.67

^a^ X‐ray fluorescence (XRF), X‐ray diffraction (XRD), fourier transform infrared spectroscopy (FTIR), thermogravimetric analysis (TGA/DTA), particle size distribution by laser dispersion, surface area (BET method).

Moreover, the chemical composition results demonstrated variability in component concentration for the different clay samples studied, which could be explained by the fact that samples were collected from different sites in São Paulo State. Specifically, XRD characterization of each clay revealed the presence of kaolinite and illite as the predominant mineralogical phases. Moreover, higher concentration of silicon as the mineral was detected in all samples. The elemental composition was similar across clays, with silicon (Si), aluminum (Al), iron (Fe), and potassium (K) being the most abundant elements. The presence of chemical bonds O—H, Si—O and Al—OH—Al is confirmed by FTIR and TGA/DTA. Knowledge of particle size distribution features of the raw materials is essential for the right pre‐formulation steps of cosmetic and pharmaceutical products. Based on the literature [48] the powder particle size distribution applicability can vary. Finer powders have higher skin adhesion and provide better softness when applied on skin. The average particle diameter ranged from 3.6 to 24.1 (clay I 3.6 μm; clay II 24.1 μm; clay III 6.9 μm; clay IV 9.7 μm). Concerning the surface area, clay sample III showed the highest value ranging around 38.57 m^2^/g, followed by clay IV (30.67 m^2^/g), clay I (28.74 m^2^/g) and clay II (22.80 m^2^/g). From the point of view of cosmetology, powder absorption properties are required for the retention of skin oiliness, thus contributing to drying and healing capacity [7]. The other materials used are particularly described in each of the specific paragraph.

### 2.2 Preparation of Formulations Containing Clays

Clays I, II, III, and IV were incorporated in two hydrophilic vehicles: (i) a nonionic emulsion and (ii) hydrophilic gel, as described below. The non‐ionic emulsion (Table 2A) was prepared following the traditional emulsification method. The aqueous phase and the oily phase were prepared separately and heated to approximately 80°C. Once both phases reached the target temperature, the aqueous phase was slowly poured into the oily phase under vigorous and continuous stirring. The emulsion was maintained under stirring until cooled to approximately 40°C. At this point, each clay (Clays I–IV) was incorporated at the concentration of 1.0% w/w. The hydrophilic gel (Table 2B) was prepared as follows: methylparaben was dissolved in hot water (~80°C). In parallel, BHT and propylparaben were dissolved in sweet almond oil and heated to 40°C. These solutions were added to a previously prepared dispersion of 0.5% of ammonium acryloyldimethyltaurate/VP copolymer in water. The mixture was left for 24 h until the formation of a homogeneous gel. The clays at the concentration of 1% w/w were incorporated into the gel after a 24‐h equilibration period at room temperature. The list of ingredients of the selected model formulations is shown in Table 2. Moreover, no chelating agents (e.g., EDTA) were added to the formulations. This choice was dictated to assess the intrinsic stability and rheological behavior of the clay‐containing systems without the influence of additional stabilizers and to isolate more clearly the specific contribution of the clays to the overall properties of the formulations.

**TABLE 2:** List of the selected formulations and information related to composition A: Non‐ionic emulsion; B: Hydrophilic gel.

A			B		
Non‐ionic emulsion			Gel		
INCI	Function	(%)	INCI	Function	(%)
EmulsifyingWax NF	Emulsifying	7.0	Ammonium Acryloyldimethyltaurate/VP Copolymer	Viscosity controller	0.5
Glyceryl Stearate	Emulsifying	3.0	Sweet Almond Oil	Emollient	1.5
Isodecyloleate	Emollient	2.0	BHT	Antioxidant	0.05
Diisopropyl adipate	Emollient	2.0	Propylparaben	Preservative	0.1
MacadamiaTernifoliaNutOil	Emollient	0.5	Methylparaben	Preservative	0.1
BHT	Antioxidant	0.05	Clay (I, II, III or IV)	Active	**1.0**
Propylparaben	Preservative	0.1	Water	Vehicle	Qsp
Propylene Glycol	Humectant	2.0			
Imidazolidinyl Urea	Preservative	0.6			
Methylparaben	Preservative	0.1			
Peg—10 Sorbitan	Surfactant	0.5			
Clay (I, II, III or IV)	Active	**1.0**			
Water	Vehicle	qsp			

*Note:* The bold formatting in the Table is intentional and serves to emphasize the table title and the percentage of clay, which corresponds to the active ingredient in the formulation.

Abbreviation: INCI, International Nomenclature of Cosmetic Ingredient.

### 2.3 Physico‐Chemical Characterization of Formulations

The physico‐chemical characterization and stability of formulations was performed according to International Conference on Harmonization of Technical Requeriments for Registration of Pharmaceuticals for Human (ICH, 2001). The formulations were stored in double‐walled bottles at room temperature (20°C ± 2°C), hothouse (Tecnal model TE‐393/1) at 45°C ± 2°C and refrigerator (Continental model 470) at 2°C ± 2°C, during a period time of 90 days. Further details about the organoleptic characteristics, pH measurements, as well as the determination of the acid index were reported in the Supporting Information.

#### 2.3.1 Particle Size Distribution Measurements

The droplet size of gels and emulsions was measured by Laser Diffraction (LD) particle size analysis with a Mastersizer 3000 (Malvern Instruments, France). The technique is based on the measurement of the intensity of light scattered as a laser beam passes through a dispersed particulate sample with the wavelength of the beam at 650 nm and a LED at 405 nm. The Mie scattering theory was used, and the continuous phase was ultrapure water. The average size ranges from 0.01 to 3000 μm. An aliquot of the sample was directly added to the humid unit for the analysis, under stirring, containing 150 mL of distilled water in order to obtain an obscuration level between 1% and 8%. All measurements were done in triplicate on days 0 and 90; the values reported were the average of the three measurements.

#### 2.3.2 Spreadability Assessment

Spreadability was assessed according to the method described by Knorst and Borghetti [49]. A circular glass mold plate (20 cm in diameter; 0.2 cm thick) with a central hole (1.2 cm in diameter) was placed over a glass support plate (20 × 20 cm). A known volume of the sample was deposited into the central orifice, after which the mold was carefully removed. A glass plate of known weight was then placed on top of the sample. After 1 min, the diameter of the spread area was measured. Additional glass plates were successively added at one‐minute intervals to apply increasing weight. Spreadability (*Ei*) was calculated as a function of the applied weight using the following Equation (1):

(1) Ei=d2*Π4

where *Ei* represents the sample spreadability for a given weight *i* (mm^2^); *d* is the mean diameter of the spread area (mm) and *π* = 3.14. Each measurement was performed in triplicate, and results are expressed as the mean value.

#### 2.3.3 Viscosity and Rheological Behavior

The viscosities of the formulations were determined using a rotational viscometer (Marte, model MDV‐20) *spindle* 4. The *spindle* rotation speed values were 1, 1.16, 1.33, 1.5, and 1.66 s^−1^ for emulsions, while 0.83, 1, 1.16, 1.33, and 1.5 s^−1^ were used for gels, for increasing and decreasing temperatures at 20°C ± 2°C, and within a torque range of 20% to 90%. The viscosity and shear rate values were used to determine the consistency index (*k*)—defined as the apparent viscosity at a shear rate of 1 s^−1^—and the flow behavior index (*n*) based on the Power Law model (Equation 2). These parameters are widely applied in the rheological characterization of semisolid cosmetic formulations, such as creams, gels, and emulsions, as they provide relevant information on product stability, texture, and application performance. The data were fitted using the software GraphPad Prism, version 6.01 (La Jolla, CA, USA).

(2) $$\eta =k\;{\dot{\gamma }}^{n-1}$$

where *η* represents the apparent viscosity (Pa·s); *γ* is the shear rate (s^−1^); *k* is the consistency index (Pa·s^*n*^); *n* is the flow behavior index (dimensionless).

The consistency index (*k*) is a key rheological parameter used to characterize the viscosity and flow resistance of cosmetic or pharmaceutical formulations, such as creams, gels, or suspensions. It helps to understand how the product behaves under different application conditions, influencing spreadability, stability, and sensory attributes. To determine k, rheological tests are performed to measure the shear stress (*τ*) and shear rate (*γ̇*) over a range of values. The data typically follow the power‐law model:

(3) $$\tau =k\cdot \dot{\gamma }ⁿ$$

where *τ* = shear stress (Pa); *γ̇* = shear rate (s^−1^); *k* = consistency index (Pa·sⁿ); *n* = flow behavior index (dimensionless). The parameter *n* indicates whether the formulation is: Shear‐thinning (pseudoplastic): *n* < 1 or Shear‐thickening (dilatant): *n* > 1. Taking the logarithm of both sides gives:

(4) logτ=logk+ηlogγ

By plotting the data log *τ* versus *γ*˙ using specialized software for data plotting and regression analysis, a straight line is obtained. The slope of this line corresponds to the flow behavior index *η* and the intercept corresponds to the logarithm of the consistency index (log *k*). The consistency index 𝑘 is then calculated as:

(5) $$k=10^{\log k}$$

The physical stability of the cosmetic formulations was evaluated through variations in the consistency index (*k*) over time. Initial and final consistency values were determined and compared, as shown in Equation (6).

(6) $$\text{Stability consistency}\;\left(\%\right)=\left(\text{final value}/\text{initial value}\right)\times 100$$

Formulations that presented values of stability (%) within the range of 90%–110% were considered stable.

### 2.4 Cell Viability Assay

#### 2.4.1 Preparation of the Extraction Solution of Clays

The determination of the cytotoxicity and the biocompatibility of the clays was performed at the UCS Laboratory of Genome, Proteomics and DNA Repairs by indirect contact analysis, according to the methods described in ISO 10993‐5‐2009‐2 (International Standard, 2009). The extraction solution of each clay was prepared using 1000 μg/mL of clays I, II, III, and IV, and DMEM culture medium (Dulbecco's Modified Eagle Medium) supplemented with 10% fetal bovine serum (FBS) and 1% penicillin/streptomycin (P/S). Subsequently, each extraction solution of clays was sonicated for 1 min in an ultrasonic bath (Unique USC‐1400A) at 37°C with a frequency intensity of 40 KHz (kilohertz). After sonication, the culture medium was incubated in contact with the clay samples for 24 h at 37°C in 5% CO_2_ in order to obtain the extraction solution. The obtained extraction solutions were then filtered with 0.45 and 0.22 μm membranes for performing the assays.

#### 2.4.2 Evaluation of Cell Viability in Keratinocytes

Cell viability was assessed through the indirect method of the MTT, as described in the standards ISO 10993‐05 (2009) and ISO 10993‐12 (2012). This method is based on the reduction of MTT (3‐(4.5‐dimethylthiazol‐2‐yl)‐2.5‐diphenyltetrazolium bromide) by the mitochondrial enzyme dehydrogenase, in the formation of crystals of formazan. The MTT assay was used to investigate mitochondrial function as described by Mossman [50]. HaCat cells (keratinocytes) were seeded at a density of 1 × 10^5^ cells/mL^−1^ in 100 μL of culture medium DMEM (Dulbecco's Modified Eagle Medium) supplemented with 10% fetal bovine serum (FBS) and 1% penicillin/streptomycin (P/S). After 48 h, the cells were treated with the extraction solution of clays I, II, III, and IV at concentrations of 62.5, 125, 250, 500, and 1000 μg/mL for 24 and 48 h. For the negative control, DMEM medium (10% FBS and 1% P/S) was used. For the positive control, an agent that causes cell death was used, DMEM medium (10% FBS and 1% P/S) with 5% DMSO (dimethyl sulfoxide). The samples were incubated at 37°C in 5% CO_2_ at the same time as above. The medium was removed and 1 mg/mL of MTT in FBS‐free medium and P/S was added to the wells. Plates were incubated at 37°C for 2 h in a humidified atmosphere with 5% CO_2_. Subsequently, the MTT solution was withdrawn and the formazan crystals were dissolved in 100 μL of DMSO. Spectrophotometric reading at 570 nm was performed on a microplate reader (Max190 spectra, Molecular Devices), and the results were expressed as a percentage of viability. The absorbance of the negative control represented 100% viability, and the values of the treated cells were calculated as a percentage of the control. Alterations in cell viability analyzed by the indirect method were observed and documented after 24 and 48 h of exposure to the treatment of the sample.

#### 2.4.3 Morphological Alteration Test

The morphology of HaCat cells was observed at 24 and 48 h after exposure to different concentrations (62.5, 125, 250, 500, and 1000 μg/mL) of extraction solution of clays using the Giemsa staining method [51]. After treatment, the cells were fixed with May‐Grunwald‐Giemsa for 1 min and stained with Giemsa staining solution 1:10 (v/v) for 10 min. Afterwards, the cells were observed in an inverted microscope [51] equipped with a digital microscope camera (Leica DFC320, Wetzlar, Germany). Representative images were taken at 200X magnification for each condition directly from a 96 multiwell plate.

### 2.5 Evaluation of In Vivo Dermal Irritability of Hydrophilic Gel Formulations

Clays I, II, III, and IV were incorporated into a hydrophilic gel as reported in Table 1 and tested to evaluate the in vivo dermal irritability. This study was conducted in accordance with the principles of the Declaration of Helsinki, applicable regulatory requirements, including CNS Resolution No. 466/12, and with Good Clinical Practice (Document of the Americas and ICH E6: Good Clinical Practice). The dermic irritability test was performed by the company Allergisa (São Paulo/SP Brazil) with the hydrophilic gel formulations containing the incorporated clays in order to confirm the absence of dermal irritation and skin sensitization of a product applied on the skin under maximized conditions. The area of application and the amount of product applied were controlled, and the whole test was supervised by a dermatologist. The test was performed in 53 volunteers using the patch test method. The volunteers were selected according to the following inclusion criteria: intact skin in the test region, agreement to adhere to the procedures and requirements of the test, age from 18 to 70 years, both sexes, and phototype (Fitzpatrick): I to IV. Healthy research participants, that is, without skin marks in the experimental area that would interfere with the evaluation of possible skin reactions (pigmentation disorders, vascular malformations, scars, increased hairiness, large numbers of ephelides and nevi, sunburns), without active dermatoses (local or disseminated) that could interfere with the results of the study. This study followed the general principles of the Human Repeated Insult Patch Test (HRIPT), a well‐established method for evaluating dermal irritation and sensitization potential in humans [52, 53, 54]. The product was distributed over a disc of filter paper of the contact test properly identified, and the control, in another disc, was also identified; both were fixed in the scapular area and in the right or left back of the study participants. The applications were performed three times a week for three consecutive weeks (induction period). During the week, the product kept in touch with the skin for 48 h between applications and for 72 h on the weekends. After each removal of the product, the area was evaluated, and the product was always reapplied on the same area. After the induction period, there was a rest period of 10 days when no patch was applied. After the rest period, the product and control were applied again, remaining in contact with the skin for a period of 48 h (challenge period). The product was removed, and the area was evaluated after nearly 30 min and 24 h. The study participants were supervised by a dermatologist throughout the study.

### 2.6 Statistical Analysis

Statistical significance was assessed using one‐way analysis of variance (ANOVA) with the multiple mean comparison test (Tukey) to assess the different statistics. ANOVA residues were tested for normality with the D'Agostino–Pearson test. The significance level was set at *p* < 0.05 using the Statistical Package for the Social Sciences (SPSS, version 19.0) for Windows.

## 3 Results and Discussion

### 3.1 Particles/Droplets Diameter Measurements

The choice of nonionic emulsions and hydrophilic gels as vehicles for clay incorporation was driven by their relevance in dermatological and cosmetic applications. Nonionic emulsifiers are widely used due to their lower irritancy and better tolerability compared to ionic surfactants, making them suitable for sensitive skin and for assessing the biocompatibility of new ingredients [55, 56, 57]. These systems also offer formulation flexibility and good physical stability, which are key characteristics in the development of safe and effective topical products [58, 59]. On the other hand, hydrophilic gels are appreciated for their non‐occlusive, refreshing properties and ease of application, making them especially suitable for oily or irritated skin [60, 61, 62]. These two vehicles represent widely accepted delivery systems in dermocosmetic science [63], providing a relevant framework to assess the physicochemical behavior and dermatological safety of novel clay‐based formulations. The diameter of the particle size of gels and emulsions formulations containing clays is presented in Table 3.

**TABLE 3:** Diameter of the particle size of gels and emulsion formulations containing clays.

Samples	Time/diameter *d* (0.5) μm			
	Initial	TA 90	*G* 90	*E* 90
*G*‐*P*	36.75	104.02	98.82	63.44
*G*‐I	51.10	81.91	76.10	71.37
*G*‐II	31.92	49.21	46.11	40.89
*G*‐III	21.40	42.77	40.51	41.23
*G*‐IV	18.83	40.09	37.78	36.83
*E*‐*P*	13.82	25.07	20.21	43.60
*E*‐I	10.47	16.90	17.78	11.52
*E*‐II	10.47	18.53	19.41	11.53
*E*‐III	9.95	15.40	19.76	10.02
*E*‐IV	9.40	17.71	15.76	21.09

Abbreviations: *E* 90, diameter values in hothouse (45°C ± 2°C) after 90 days of storage; *E*, emulsion; *G* 90, diameter values in refrigeration (2°C ± 2°C) after 90 days of storage; *G*, gel; I–IV, type of clay; Initial, diameter values after the preparation of formulation; *P*, control; TA 90, diameter values under room temperature (20°C ± 2°C) after 90 days of storage.

Our results showed that the addition of clays reduced particle size of all gel samples, excepting from the gel within clay I, where the size of such formulation increased compared the control gel. After 90 days of storage, all gel samples containing clays showed an increase in the particle diameter compared to the initial values measured at day 0 (Table 3). This may be justified by the swelling of the clays due to the presence of water in these formulations, promoting an increase in particle diameter [25]. A similar trend was observed for emulsions. Initially, the particle diameters of emulsions decreased with the addition of clays and after 90 days of storage all samples showed a slightly increase in particle diameter compared to the initial values (Table 3). Specifically, emulsions containing clays I, II, III and IV showed close values of diameter at day 0 and after 90 days (Table 3). Moreover, it was observed that both controls (gel or emulsion without clays) presented values of diameter higher than those observed in the other samples, indicating that the addition of the clays caused a decrease in the particle diameter for all samples tested. Further, it is important to note that gels have larger diameters than those of emulsions at the end of 90 days of storage in the tested conditions (Table 3). However, emulsions may become unstable due to processes causing progressive particle growth and non‐uniform size distribution, leading to droplet coalescence and phase separation. For this reason, it is important to evaluate the particle size over time in order to determine the stability of the system [64]. It was observed that after preparation, emulsions initially showed a monomodal distribution for all samples. The standard emulsion and the emulsions within clays I and III showed a similar distribution profile after the 90 days of storage under different conditions, representing an initial stability of such systems. On the other hand, emulsion with clay II showed a bimodal distribution after the 90 days of storage at all tested temperatures (2°C; 20°C and 45°C), while emulsion with clay IV presented bimodal distribution at 2°C and at 45°C, but remained monomodal at 20°C. The bimodal distribution demonstrated by such emulsions indicates instability in the system. This may be caused by the interactions of the clays with the emulsions or it may be attributed to the storage time in the tested conditions, resulting in reduced stability for these two emulsions. However, gels exhibited monomodal distribution after preparation and the same profile was registered after 90 days of storage in different conditions for all samples tested. Although the particle size increased over time for all samples, their monomodal distribution was uniform, resulting in higher stability in gel systems compared to emulsions. Furthermore, control (gel without clays) kept all organoleptic properties stable throughout the testing period, while control exhibited instability in the system emulsion resulting in an alteration of coloration and phase separation at 45°C ± 2°C. Indeed, this can be explained by the fact that the addition of clays in the emulsions may have contributed to the formation of the Pickering emulsions (PE), typically emulsions stabilized by a thin solid layer, either organic or inorganic compounds [65, 66]. Pickering emulsions are able to maintain the droplets of the outer phase in high concentrations, which means that they can maintain the emulsification process under balance and allow the system re‐dispersion [67]. Notably, the clays are inorganic compounds and they can lead to the formation of PE. However, more detailed and specific characterization studies should be performed for our systems in order to confirm this hypothesis. Moreover, it is important to point out that clays such as bentonite, magnesium aluminum silicate and kaolin, form gels by flocculation. Specifically, they are hydrated silicates of aluminum (or aluminum and magnesium), whose structure of the crystals has a structure resembling flat plates. The flat part or “face” of particles present negative charges due to the oxygen atoms, and the edges of the plates have positive charges due to Al/Mg atoms. As a result of the electrostatic interactions between the “face” and the “edge” of different particles, a gel structure, usually known as “house of cards flock” structure was formed [25]. This may be a possible explanation for the higher stability of gels than emulsions after the addition of clay with respect to particle size distribution. Furthermore, the stability of formulations was also influenced by clay properties. Particle size distribution and organoleptic characteristics varied according to the type of clay used. Gels maintained monomodal distributions and stable organoleptic features over 90 days, reflecting the stabilizing effects of clay flocculation and electrostatic interactions. In contrast, emulsions containing certain clays (e.g., II and IV) displayed bimodal particle distributions under some storage conditions, indicating partial instability. Overall, these results emphasize that the physicochemical differences among clays are crucial for defining the final performance and stability of gel and emulsion formulations.

### 3.2 Determination of Spreadability

**FIGURE 1:**
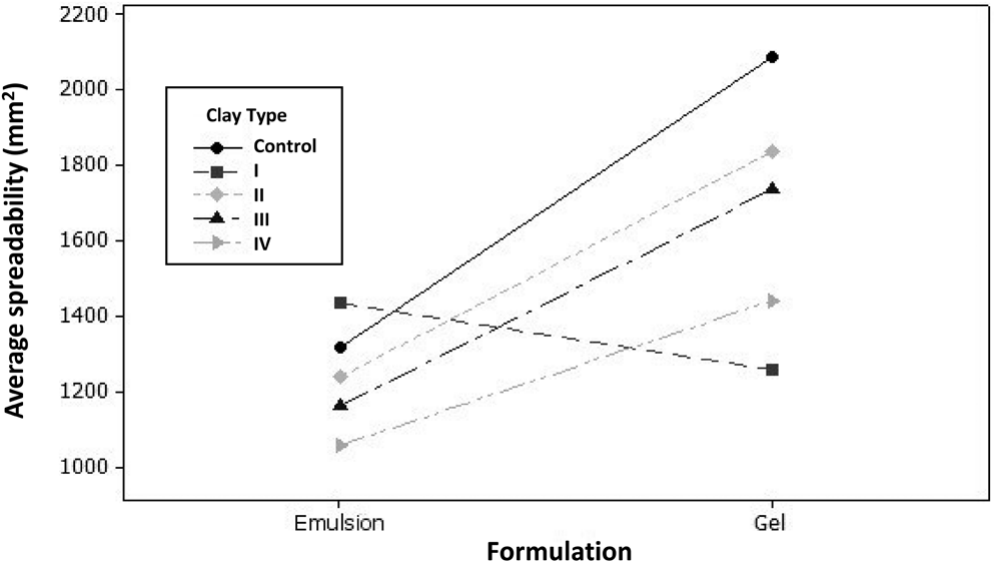
Spreadability values of tested formulations.

The spreadability of both formulations within four different types of clay was analyzed and illustrated in Figure 1.

Our results showed that the spreadability of gels was higher than those measured for emulsions (2200 vs. 1500 mm^2^) (Figure 1). This may be attributed to the interactions between the components of gels compared to the emulsions. However, it is important to highlight that the specific ingredient compositions of our semi‐solid systems (emulsions and gels) may alter the rheological properties of such formulations, making direct comparison between gels and emulsions difficult. Moreover, the spreadability analysis revealed that clay I showed a different behavior compared to other clays (Figure 1). This may be due to the presence of kaolinite in its composition, according to XRD measurements [46]. So, this could be explained by interactions between clay I and the vehicles, leading to (i) increased spreadability of the emulsion containing clay I and (ii) decreased spreadability of the gel within clay I. Notably, after 90 days of storage under different conditions, almost all clays decreased the spreadability of both systems (emulsion and gels), but this aspect was not observed for the emulsion containing clay I, which showed the opposite trend. Additionally, it was noted that both gels (with and without clays) exhibited higher spreadability than emulsions. Similarly, clay type I exhibited a different behavior: lower spreadability values were found for gel within clay I compared to those measured for the emulsion containing clay I. Spreadability is an important parameter for cosmetic applications, affecting the efficacy and the acceptability of the product by the consumer. The viscosity of formulations is significant in their application since it is inversely correlated to the spreading: on the one hand, a highly viscous formulation shows low spreadability, while on the other hand, low viscosity eases application [68]. In fact, a formulation with low spreadability leads to a non‐homogeneous distribution of the product on the skin surface, affecting the applied dose, the efficiency of skin penetration of the active ingredients, and consumer acceptability [69, 70]. It is important to point out that no correlation was found between spreadability and viscosity in both systems containing clays; however, increases or decreases in these two parameters were observed depending on the test conditions. The spreadability of formulations was clearly influenced by the physicochemical characteristics of the incorporated clays. Clay I showed a lower particle size than the other clays, measuring 3.6 μm. Smaller particles are expected to have a greater surface area, which facilitates better dispersibility within the gel. Furthermore, since clay I is mainly composed of kaolinite, the effect of this compound on gel spreadability is important. Kaolin particles tend to aggregate and form a three‐dimensional network within the formulation. In gels, this network reinforces the existing gel structure, making it much denser and more rigid, thereby severely restricting flow and spreadability. In emulsions, the same effect occurs due to the particle network, which increases viscosity; however, it is less pronounced because the dispersed phase (oil or water) can provide some lubrication, slightly mitigating the overall rigidity compared to a gel. It was observed that gels containing clays with a larger particle size (clay II) exhibited a lower reduction in spreadability. Another important factor to consider is that clay I consists exclusively of kaolinite, whereas the other clays have a mixture of kaolinite, smectite, and illite. This mixed composition had a synergistic effect, resulting in a lower reduction in gel spreadability compared to the control. These observations demonstrate that the type and properties of clay, including mineral composition, particle size, and surface charge, play a critical role in defining the ease of application of semi‐solid formulations and must be considered during formulation development.

### 3.3 Evaluation of Viscosity

**FIGURE 2:**
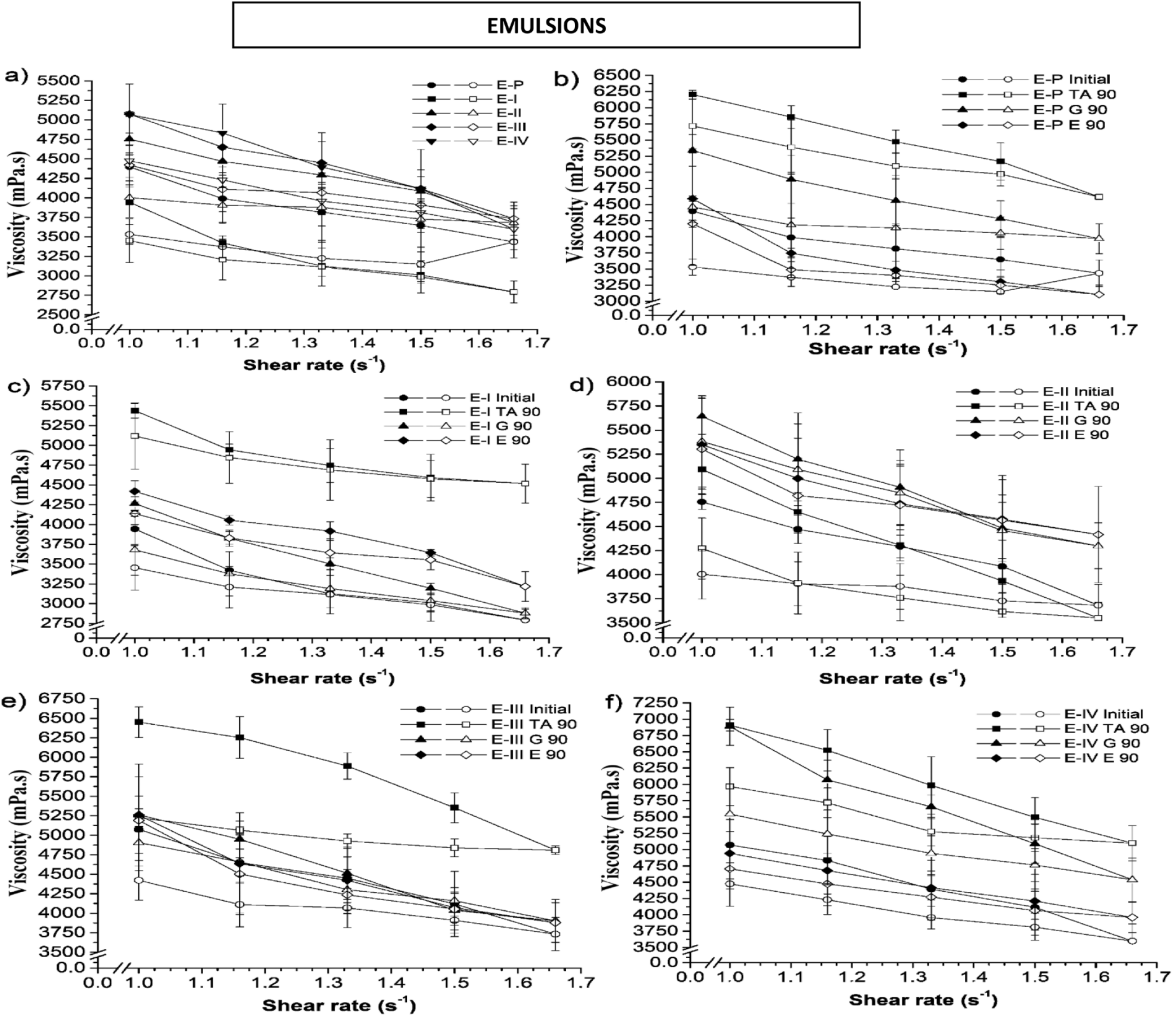
Viscosity of emulsion samples containing clays at: (a) all at zero time, (b) control, formulations with (c) clay I, (d) clay II, (e) clay III, (f) clay IV, under different storage conditions. (*E* 90, viscosity values in hothouse (45°C ± 2°C) after 90 days of storage; *E*, emulsion; *G* 90, viscosity values in refrigeration (2°C ± 2°C) after 90 days of storage; I–IV, type of clay; Initial, viscosity values after the preparation of formulation; *P*, control; TA 90, viscosity values under room temperature (20°C ± 2°C) after 90 days of storage). Fulfilled symbol: departure; Empty symbol: return. The data points represent the mean ± the standard deviation of three measurements.

**FIGURE 3:**
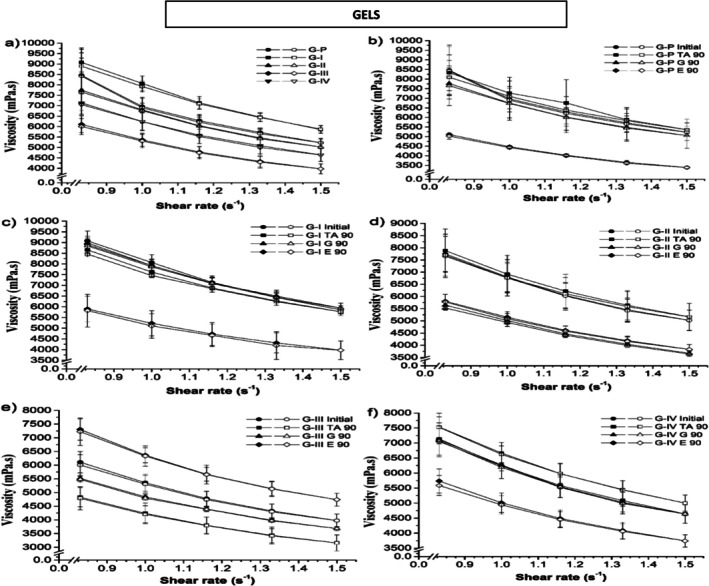
Viscosity of gel samples containing clays at: (a) all at zero time, (b) control, formulations with (c) clay I, (d) clay II, (e) clay III, (f) clay IV, under different storage conditions. (*E* 90, viscosity values in hothouse (45°C ± 2°C) after 90 days of storage; *G* 90, viscosity values in refrigeration (2°C ± 2°C) after 90 days of storage; *G*, gel; I–IV, type of clay; Initial, viscosity values after the preparation of formulation; *P*, control; TA 90, viscosity values under room temperature (20°C ± 2°C) after 90 days of storage.). Fulfilled symbol: departure; Empty symbol: return. The data points represent the mean ± the standard deviation of three measurements.

The physical appearance, consumer perception, spreadability, and flow of semi‐solid formulations such as gels and emulsions can be altered due to the change in the viscoelastic properties of such systems [71]. The rheological properties of formulations are reported in Figures 2 and 3. The evaluation of the rheological behavior as a function of temperature is a key approach for obtaining information on the physical stability and consistency of the product. According to Florence and Attwood [72], the solubility of nonionic emulsifiers changes as the temperature increases, and temperatures over 70°C are able to destabilize the majority of emulsions. Moreover, low temperatures lead to ice formation, causing the emulsion's breakdown through disruption of the interfacial film. The viscosity of both gels and emulsions was influenced by temperature, reflecting the physical and structural characteristics of the formulations. For emulsions, increasing temperature can reduce the viscosity due to decreased solubility of nonionic emulsifiers and increased molecular mobility, potentially leading to partial destabilization or coalescence of dispersed droplets. In gels, the viscosity changes with temperature are less pronounced, as the flocculated networks formed by clays maintain structural integrity and resist thermal fluctuations. At low temperatures, emulsions may experience slight increases in viscosity due to water crystallization or restricted molecular mobility, while gels remain stable because of their gel matrix. These temperature‐dependent behaviors are critical for predicting product performance under storage and application conditions, as they influence spreadability, ease of application, and long‐term stability. Understanding these mechanisms allows the design of formulations that retain consistent rheological properties across the range of expected temperatures.

**FIGURE 4:**
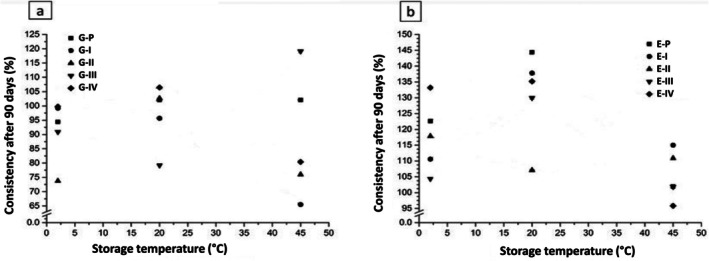
Results of the consistency of the formulations (a): gels; (b) emulsions (*E*, emulsion; *G*, gel; I–IV, type of clay; *P*, control.).

Rheological measurements were performed using a rotational rheometer, with shear rates selected to mimic low‐shear conditions relevant to the application of topical gels and emulsions. The torque values were monitored to ensure they remained within the operational range of the instrument, and repeated measurements confirmed the reproducibility of the data. Although low shear rates were applied, they are appropriate for evaluating the flow behavior of semi‐solid systems under conditions similar to spreading on the skin. The consistency of the results across replicate measurements validates the reliability of the rheological data and supports the interpretation of pseudoplastic flow and viscosity trends in both gels and emulsions. It was observed that the emulsions containing clay and the control emulsion (without clays) presented non‐Newtonian behavior and pseudoplastic flow (Figure 2). Notably, pseudoplastic flow is characterized by a decrease in apparent viscosity, as the applied shear stress increases. This type of behavior is expected in many pharmaceutical formulations [73, 74]. The apparent viscosity increases at low shear stress is necessary to limit the mobility of the dispersed phase. The viscosity of the emulsions can be altered by the volume and concentration of the dispersed phase, the nature of the emulsifying agent and the emulsifying conditions. Furthermore, above the critical flocculation concentration, emulsions become highly viscous with a pronounced tendency towards a non‐Newtonian behavior [75]. The emulsions should demonstrate free flow with low viscosity when they are stirred, and at high shear stresses provided these alterations are reversible after a period of rest, such systems can delay coalescence or creaming phenomena [76]. The rheological curves of emulsions exhibited higher variability compared to gels, which can be explained by the intrinsic properties of the systems. Emulsions containing clays showed a non‐Newtonian pseudoplastic behavior, making apparent viscosity sensitive to shear rate. In addition, the different physicochemical characteristics of the clays, including particle size, surface charge, and flocculation tendencies, contributed to variations in flow behavior. Small differences in preparation, storage conditions, or particle interactions further amplified this variability. Despite these fluctuations, the overall trends were consistent and reproducible across repeated measurements, and the observed differences accurately reflect the influence of clay type on emulsion rheology. In contrast, gels maintained more uniform viscosity and monomodal particle distribution, indicating higher stability under the tested conditions. The rheological data were modeled using the Power Law equation to obtain the flow behavior index (*n*) and consistency index (*k*). For both gels and emulsions, the flow behavior index was less than 1, confirming pseudoplastic (shear‐thinning) behavior. Emulsions generally exhibited slightly lower n values than gels, indicating higher shear‐thinning tendencies, likely due to the interactions between the dispersed droplet and the influence of clay particles. Gels maintained higher *n* values, reflecting more uniform structure and lower sensitivity to shear. These results are consistent with the observed viscosity trends and stability profiles, highlighting the influence of clay type and vehicle on the rheological properties. The flow behavior index thus provides a quantitative measure of pseudoplasticity and complements the qualitative interpretation of the rheological curves. Furthermore, the rheograms obtained during the analysis exhibited the typical behavior of a pseudoplastic system, in which viscosity decreases with increasing shear rate. At day 0, higher viscosity values were registered for emulsions containing clays II, III and IV compared to the standard emulsion (Figure 2), while emulsions containing clay I showed lower viscosity compared to the standard emulsion. After 90 days of storage, an increase in viscosity was observed for emulsions containing clays I, III and IV compared to the values measured on day 0 for the same formulations. Yet, the emulsion with clay II showed a decrease in viscosity at 20°C ± 2°C and an increase at 2°C ± 2°C and 45°C ± 2°C. However, the control emulsion showed a decrease in viscosity at 45°C ± 2°C and an increase at 20°C ± 2°C and 2°C ± 2°C. Concerning rheological properties of gels, our data demonstrated that gels containing clays and the control gel (without clays) showed non‐Newtonian behavior and pseudoplastic flow (Figure 3). Such parameter is often preferred for gels that are intended for cosmetic formulations [77, 78, 79]. Our measurements reported that lower viscosity values were registered for gels containing clays II, III and IV at day 0 compared to those analyzed for the control gel (Figure 3), while gels containing clay I exhibited higher viscosity than control. It is important to underline that the gels showed the opposite trend compared to those observed for emulsions. After 90 days of storage in different conditions, the control gel and the gel containing clay II showed an increase in viscosity at 20°C ± 2°C and a decrease at 2°C ± 2°C and 45°C ± 2°C. The gel samples containing clay I showed a decrease in viscosity at 45°C ± 2°C, while the values at 2°C ± 2°C and 20°C ± 2°C were similar to those measured at day 0. There was an increase in viscosity for the gel with clay III at 45°C ± 2°C, while a decrease was observed at 2°C ± 2°C and 20°C ± 2°C. Moreover, for the gel containing clay IV viscosity increased at 20°C ± 2°C, decreased at 45°C ± 2°C and remained similar to day 0 at 2°C ± 2°C. Further, the viscosity of the gels was visually observed to be slightly higher than that of emulsions. It is important to note that the addition of the clays to formulations, reduces the viscosity of such systems. Additionally, the storage temperature ranging around 45°C ± 2°C reduced the viscosity measurements of the systems compared to those values obtained at 20°C ± 2°C and 2°C ± 2°C. However, both tested formulations (gels and emulsions) presented mean values of apparent viscosity over 4000 mPa·s, considering the shear rate of 1 s^−1^. Our viscosity values are in line with the study of [80] where they tested semi‐solid systems containing clays extracted from the seabed. Similarly, our data were in agreement with Modabberi et al. [81] who studied bentonite clays for pharmaceutical applications. Furthermore, the results of viscosity were modeled according to the Power Law in order to provide a consistency value (k). The stability of emulsions and gels containing clays was evaluated by comparing the initial consistency values with those calculated after 90 days of storage under different conditions. The obtained results are shown in Figure 4. Our data revealed that after 90 days of storage at temperatures of 2°C and 20°C, gels presented less variation of consistency than those measured for emulsions under the same storage conditions. An increase of consistency was observed in almost all emulsion samples, with values exceeding 100% of stability (Figure 4). On the other hand, this behavior was not registered for gel samples which showed values below 100%. However, regarding the viscosity parameter gels were more stable than emulsions.

Moreover, from the modeling, a good fit of the chosen model was obtained for the gels, while only a reasonable adjustment was achieved for the emulsions, since the determination coefficient values (R^2^) were not close to 1 for some emulsion samples. Overall, these observations demonstrate that the intrinsic physicochemical properties of each clay, including particle interactions, flocculation behavior, and swelling capacity, play a key role in determining the viscosity and stability of both gels and emulsions. Gels, generally, showed higher stability and more uniform viscosity over time compared to emulsions. These differences were mainly attributed to particle interactions, flocculation phenomena, and the swelling behavior of the clays. Clays forming flocculated networks, such as bentonite and magnesium aluminum silicate, increased the structural consistency of gels, whereas emulsions were more susceptible to changes in viscosity and particle redistribution during storage, particularly those containing clays II and IV. These findings demonstrate that the intrinsic physicochemical characteristics of clays directly affect rheological behavior.

### 3.4 Evaluation of In Vitro Cytotoxicity of Clays

**FIGURE 5:**
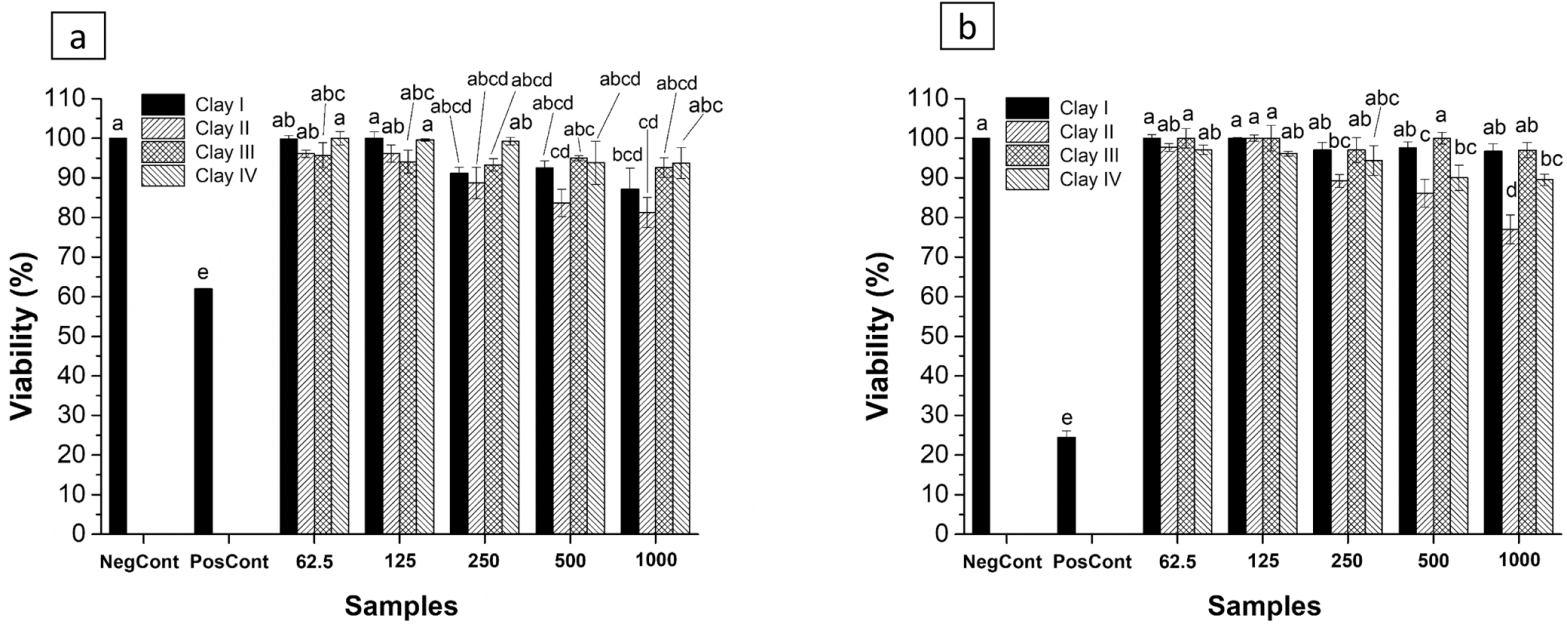
Effect of different concentrations (1–0.062 mg/mL) of clays on HaCat viability measured by MTT assay after (a) 24 and (b) 48 h exposure. Data are given as mean value ± S.D. NegCont, negative control; PosCont, positive control.

The cytotoxic effects of clays I, II, III, and IV were investigated using HaCat keratinocytes as a cell model. The cytotoxic effect of different concentrations of clays was detected through the MTT assay, a reliable test that measures mitochondrial activity and cytotoxic potential of compounds in established cell lines as well as in primary and secondary cultures [82]. The in vitro cell viability assay is shown in Figure 5.

**FIGURE 6:**
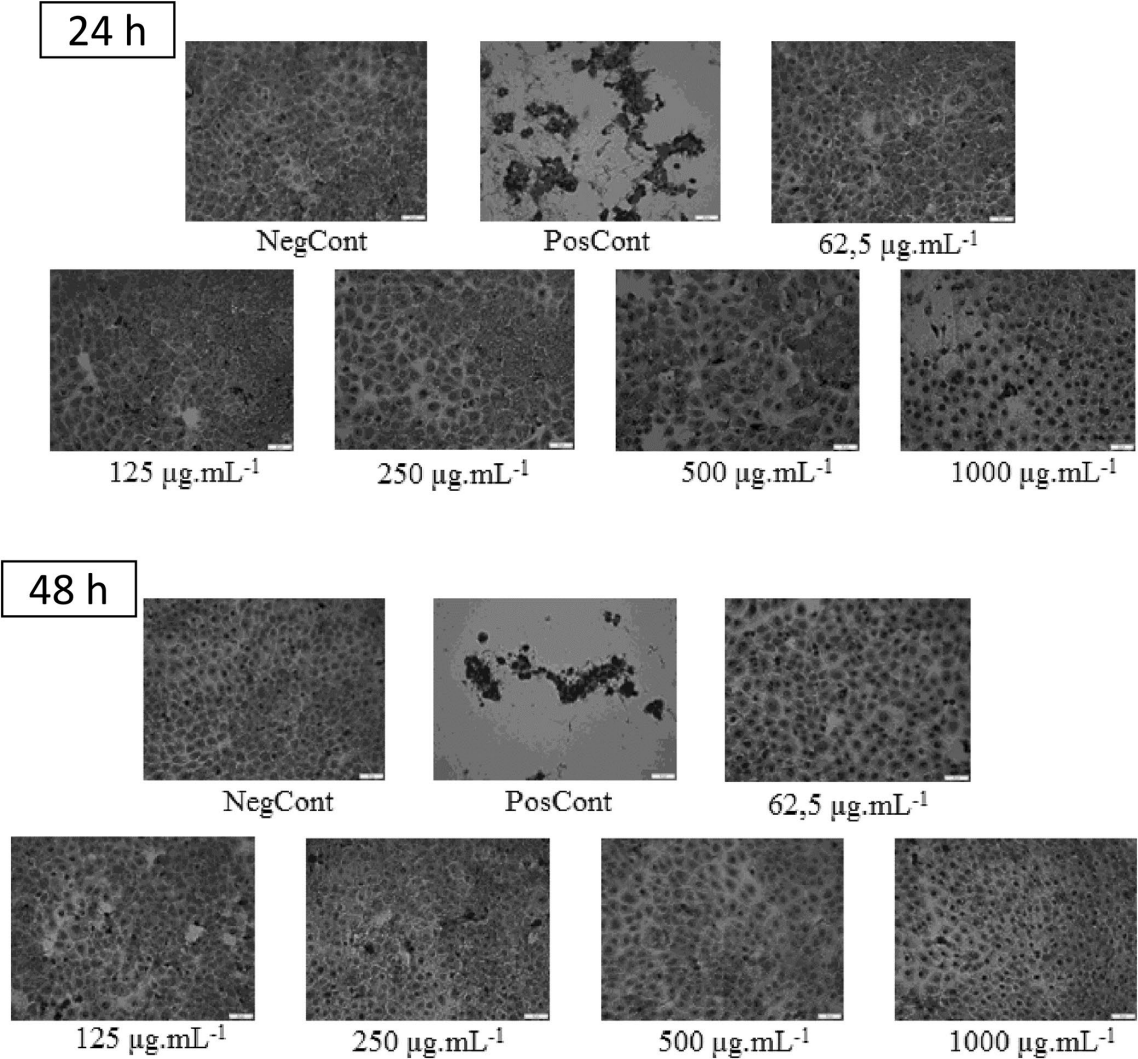
Morphological evaluation of HaCat cells after 24 and 28 h exposure to different concentrations of clays (1–0.062 mg/mL). Representative bright field images were collected at 20× magnification. NegCont, negative control; PosCont, positive control.

Results showed a dose‐dependent cellular toxicity for all compounds tested: clays I, II, III, and IV. Notably, after 24 h, a cell viability above 70% was observed for all the compounds tested, according to ISO (International Standard, 2009) [23], with the highest reduction of cell viability for clay II exposed to 1000 μg/mL (81.24%). Importantly, at higher concentrations (250, 500, and 1000 μg/mL), all tested clays did not significantly affect cell viability after 24 h and can therefore be considered non‐cytotoxic. After 48 h, a decrease in cell viability was observed for the cells treated with all clays, suggesting that exposure time can influence cell viability. Similarly, at the highest concentration (1000 μg/mL), clay II showed the highest reduction in cell viability (76.9%) compared to the other clays (89.54% and 100% of cell viability) (Figure 6). However, despite the reduction in cell viability after 48 h in the samples treated with 1000 μg/mL of clays, cell viability remained over 75%, suggesting adequate biocompatibility. According to ISO 10993‐5:2009, materials are considered non‐cytotoxic when cell viability remains above 70%, supporting the biocompatibility of the tested samples (International Standard, 2009) [83]. As mentioned in the methodology section, for the positive control, an agent that causes cell death was used, DMEM medium (10% FBS and 1% P/S) with 5% DMSO (dimethyl sulfoxide). Cell viability dropped from 60% in the first 24 h to 25% after 48 h incubation with 5% DMSO. DMSO exerts different effects on cell culture depending on the concentration and cell type. At low concentrations (e.g., 0.1%–0.5%), DMSO may not significantly impact cell viability or proliferation [84]. However, higher concentrations (e.g., 1%–5%) can lead to reduced cell viability, changes in morphology, decreasing proliferation, and affecting cellular processes like apoptosis, cell cycle, and cytokine production. When used as a positive control, DMSO helps to demonstrate the potential cytotoxic effects of a compound or treatment by showing a reduction in cell viability. Although not tested in this study, among a series of effects, DMSO can reduce the production of cytokines, such as TNF‐α and IFN‐γ. Pro‐inflammatory cytokines are known to be involved in the body's response to skin injury and irritation and play a crucial role in triggering inflammation, followed by tissue degradation and repair. The cytotoxicity of clays has been previously investigated, especially for those of natural or industrial origin, such as bentonite, kaolin, and montmorillonite. Several studies have reported that certain clays may exert dose‐dependent cytotoxic effects on keratinocytes or fibroblasts, often related to their metal content, particle size, or surface reactivity [85, 86]. For example, montmorillonite modified with organic surfactants showed significant cytotoxicity in vitro [87], whereas natural bentonite was generally well tolerated at low concentrations [88]. Our results are in agreement with those described in the literature for toxicity tests involving this type of material. A study evaluated the cytotoxicity of kaolinite aluminosilicates. Cytotoxicity of the samples was assessed using standard MTT assay protocols to evaluate the total activity of mitochondrial respiratory enzymes. Cultured cells of tumor origin, specifically human histiocytic lymphoma cells (U937), were used in the experiments. Particles with spherical and nanosponge morphologies showed no toxicity in the tested models [89]. Awad et al. [90] concluded that kaolinite and its derivatives can be safely used in biomedicine, including in the design and development of platforms for optimized diagnostics and treatment of relevant pathologies such as cancer and immunological diseases. Kaolinite can also be applied in modified drug delivery, as well as in the modulation and control of the immune response for the treatment of various diseases. Perlite, kaolin, and bentonite samples were evaluated for their cellular toxicity. These materials were applied in fibroblast cell suspension. Viability results of the powders showed that bentonite had the lowest viability, about 53%, kaolin at 70%, and perlite at 90%. Light microscopic images illustrated the viability and proliferation of fibroblast cells on perlite and kaolin surfaces, and SEM images also indicated strong cell adherence, particularly on perlite surfaces. These results support the safe use of clays in topical products, in agreement with our findings. These findings underline the importance of safety evaluation for each specific clay type intended for dermatological or cosmetic use. Morphological examination using Giemsa staining as a complement to the indirect MTT method revealed that the clays did not show significant alterations in cell morphology (Figure 6). The cells grew with a round morphology, displaying abundant well‐defined cytoplasm. Notably, after 24 h at the higher concentrations (1000 μg/mL) of clay II, cell growth was inhibited, showing substantial morphological alterations, including reduced cell number and cytoplasm condensing. These observations were also confirmed by the cytotoxicity assay. According to the morphological analyses, the clays tested in our study showed no cytotoxic activity in the parameters used.

Concerning the dermal irritability test, our results demonstrated that no volunteer showed cutaneous clinical signs related to the product. Notably, the process of primary and accumulated irritation, as well as cutaneous sensitization, was induced by the product in the tested group, suggesting the safety of such a product under tested conditions. These findings suggest a good alignment between the in vitro and in vivo results. Specifically, the low cytotoxicity observed in HaCat cells at the tested concentrations is consistent with the absence of dermal irritation or sensitization in human volunteers, supporting the overall biocompatibility of the clay‐based formulations. However, it is important to point out that the skin contact of the product can cause several types of reactions, such as immediate or cumulative irritative reactions, allergic reactions, dermatitis, systemic, and carcinogenic reactions [91]. Among the cutaneous reactions that could have occurred in the skin of the participants, the following stand out: contact dermatitis, urticaria, acne, and blemishes [92, 93]. Moreover, the assessment of the irritative and sensitizing potential of a product should consider various parameters, such as the components and concentrations of the ingredients, the amount of product applied, skin condition, mode and frequency of application, cumulative effect of the application of the product, and absorption rate of the product through the skin [38]. Generally, it is well known that some areas of the body are more susceptible to irritation than others, which may affect the skin penetration pathways of molecules or ingredients in the products [34, 94]. Skin conditions, mainly the degree of hydration of the skin, can influence the irritation potential of a substance or product [95]. However, the results of an in vivo study involving 65 women demonstrated that the transepidermal water loss did not show a direct correlation between the skin in normal individuals and the irritability of a product, suggesting that the product or active ingredients may lead to these cutaneous reactions and that the severity of irritability reactions may be attributed to the skin conditions of the individuals [37]. Additionally, a product applied on the skin surface showed high or low percutaneous absorption depending on its concentration, type of vehicle used, skin surface area, and time of contact with the skin [38]. Concerning our study, the clays were developed to be applied on the face, so it is important to ensure their safety and evaluate dermal irritability in order to assess the possibility that these products may cause irritation. The clays prepared were incorporated into the hydrophilic gel with a concentration of 1%. The dermal irritability test revealed that the clays do not induce primary or secondary irritation, confirming the safety of these products for future consumer use. Further, it is important to know that a substance can be a toxic agent, causing irritative or sensitizing reactions. This can be attributed to the exposure conditions, such as the amount of dose administered, time and frequency of exposure, and administration [38, 96]. Thus, it is important to point out that higher concentrations of clays are not exempt from causing irritative reactions or sensitization. Finally, as observed in the evaluation of in vitro cytotoxicity, the tested clays showed low cytotoxic potential, indicating that at this level the clays are not toxic. These observations were also confirmed by the in vivo test for irritability. A product with possible cytotoxic activity is more likely to cause some kind of irritative or sensitizing reaction [97].

## 4 Conclusion

Our findings demonstrate that sand‐derived clays can be successfully incorporated into gels and non‐ionic emulsions, with gels showing greater stability under tested conditions, supporting their use in cosmetic formulations. The presence of clays affects rheological properties such as spreadability, viscosity, and particle size, with clay III showing the largest surface area that enhances skin adhesion. The lack of cytotoxicity in HaCaT keratinocytes correlates with the absence of dermal irritation in humans, indicating that the sand‐derived clays used in our formulations are biocompatible and safe for topical cosmetic use. Indeed, the consistency between the in vitro and in vivo data reinforces the cutaneous safety of these formulations. Beyond cosmetics, these results suggest potential applications in therapeutic dermatology. Due to their adsorptive capacity, soothing effect, and favorable interaction with the skin barrier, such clays may be explored in formulations targeting inflammatory or seborrheic conditions such as acne, eczema, or seborrheic dermatitis. Their ability to modulate sebum, reduce irritation, and deliver active compounds makes them promising candidates for further therapeutic applications. Further studies are warranted to confirm these potential applications. Overall, clays from sand extraction residues represent safe and promising ingredients for both cosmetic and dermatological uses.

## Author Contributions

J.S.F.: investigation, writing original draft, writing – review and editing. V.S.: conceptualization, writing original draft. V.W.‐A.: conceptualization, supervision, writing original draft, writing – review and editing. C.S.C.G.: investigation. G.C.M.: data curation, writing original draft; writing – review and editing. J.A.P.H.: investigation. M.R.‐E.: investigation, resources. D.S.M.: investigation. C.P.B.: resources. All authors have read and approved the final manuscript.

## Conflicts of Interest

The authors declare no conflicts of interest.

## Supporting information


**Data S1:** Supporting Information.

## Data Availability

The data that support the findings of this study are available on request from the corresponding author. The data are not publicly available due to privacy or ethical restrictions.
