# Supplementary material for: Physicochemical Characterization and Safety Assessment of Cosmetic Gels and Emulsions Containing Sand‐Extraction Clays
Source: J Cosmet Dermatol. 2025 Oct 23;24(10):e70517. doi: 10.1111/jocd.70517 (PMC12547854; doi:10.1111/jocd.70517)
Supplement: Supplementary file 1 — Data S1: Supporting Information. [file JOCD-24-e70517-s001.docx]

**Supplementary Information**

Physicochemical characterization and safety assessment of cosmetic gels and emulsions containing sand-extraction clays

**Physical-chemical characterizations of formulations**

**Organoleptic characteristics**

The organoleptic properties assessed through visual and sensory evaluation included appearance, color, odor, and texture. After preparation, emulsions containing clays exhibited characteristic colors depending on the type of clay used, while the base emulsion (without clay) appeared white. All emulsions had a typical emulsion odor, a homogeneous appearance, and a soft texture to the touch. After 90 days of storage under various conditions, all emulsions showed color changes when stored at 45 ± 2 °C. Additionally, the emulsion containing Clay I exhibited phase separation and an altered appearance under the same temperature condition. No changes were observed under the other storage conditions.

Similarly, gels prepared with different clays showed colors corresponding to the incorporated clay, whereas the control gel (without clay) was colorless and slightly opaque. All gels had a characteristic gel odor, a homogeneous appearance, and a soft, refreshing texture. After 90 days of storage, no changes in appearance, odor, or texture were observed in any of the gel samples stored at 20 ± 2 °C or 2 ± 2 °C. However, at 45 ± 2 °C, both the control gel and the gel containing Clay IV underwent a color change, developing an intense yellow hue.

**pH determination**

The pH of the formulations was measured. The gels containing clays exhibited a pH of 6.46, whereas the emulsions with incorporated clays showed a lower pH value of 3.38. However, both emulsions—with and without clays—presented pH values below the expected range for nonionic emulsions (pH 5.0–6.0). According to (Masmoudi et al. 2005), such a decrease in pH may indicate oxidation of the oil phase, leading to the formation of hydroperoxides, or hydrolysis of triglycerides, which results in the production of free fatty acids.

**Acidity index determination**

The acid index of the macadamia oil used in the nonionic emulsion was determined to assess whether oxidative processes had occurred in the formulation. Specifically, the fatty acid content of the macadamia oil was measured through the acid index test to evaluate potential oil oxidation. An increase in the acid index may indicate intensified hydrolysis of ester components in the lipid phase, which could contribute to a decrease in the formulation’s pH (Coradi, Souza, et Borges 2017). The macadamia oil showed an acid index of 3.63 ± 0.037 mg KOH/g, which is within the limits recommended by the World Health Organization (maximum value: 4.00 mg KOH/g; CODEX ALIMENTARIUS – WHO, 2017). This result confirms that the oil did not undergo oxidative degradation and is therefore not responsible for the low pH observed in the nonionic emulsion.

**REFERENCES**

CODEX ALIMENTARIUS COMMISSION – FAO/WHO. 2017. Codex alimentarius, fats, oils and related products. Codex alimentarius, norma para los aceites de oliva y aceites de oliva Codex Stan 33-1981 (Rev.4-2017).

Coradi, Paulo Carteri, Alex Eduardo Marchi De Souza, et Monica Cristina Rezende Zuffo Borges. 2017. « <b>Yield and acidity indices of sunflower and soybean oils in function of grain drying and storage ». *Acta Scientiarum. Agronomy* 39 (2): 255. https://doi.org/10.4025/actasciagron.v39i2.31121.

Masmoudi, H., Y. Le Dréau, P. Piccerelle, et J. Kister. 2005. « The Evaluation of Cosmetic and Pharmaceutical Emulsions Aging Process Using Classical Techniques and a New Method: FTIR ». *International Journal of Pharmaceutics* 289 (1‑2): 117‑31. https://doi.org/10.1016/j.ijpharm.2004.10.020.
